# On the rearrangement of *N*-aryl-*N*-Boc-phosphoramidates to *N*-Boc-protected *o*-aminoarylphosphonates

**DOI:** 10.1007/s00706-017-2058-x

**Published:** 2017-12-01

**Authors:** Edyta Kuliszewska, Friedrich Hammerschmidt

**Affiliations:** Institute of Organic Chemistry, University of Vienna, Vienna, Austria

**Keywords:** Phosphorus compounds, Rearrangements, Amines, Carbanions, Arenes

## Abstract

**Abstract:**

Various arylamines were converted in two steps to *N*-Boc-*N*-arylphosphoramidates. LiTMP and LDA induced directed *ortho*-metalation at temperatures from −78 to 0 °C. The ensuing [1,3]-migration of the phosphorus atom with its substituents from the nitrogen to the *ortho*-carbanionic carbon atom gave *N*-Boc-protected *o*-aminoarylphosphonates. The nature of the substituent of 3-substituted phenylphosphoramidates strongly influenced the regioselectivity of phosphonate formation. A crossover experiment with a deuterated phosphoramidate proved the intramolecular course of the rearrangement. Three representative *N*-Boc-*o*-aminoarylphosphonates were deprotected to access the corresponding *o*-aminoarylphosphonic acids.

**Graphical Abstract:**

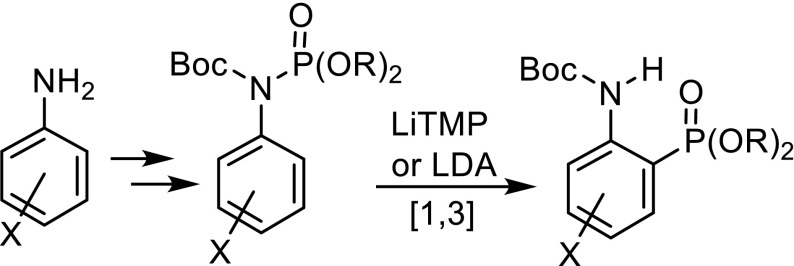

## Introduction

**Scheme 1:**
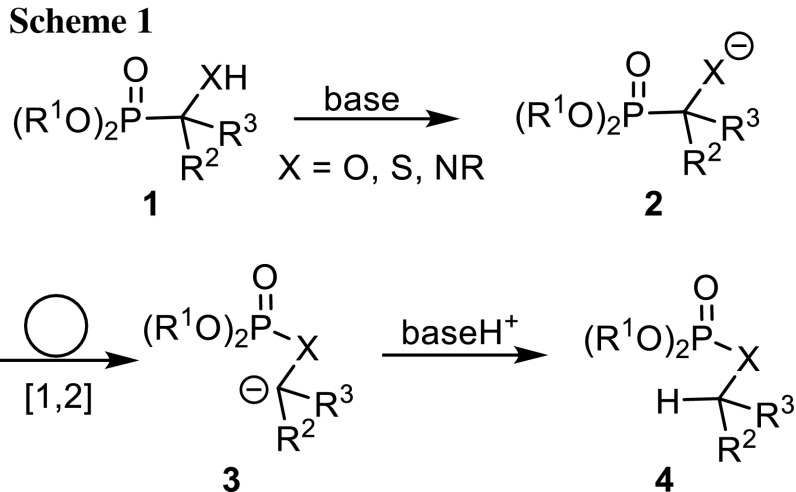
.

Phosphorus atoms in phosphonic and phosphoric acid derivatives can be induced to migrate with their substituents within molecules. The most important isomerization reactions are [1,2]- and [1,3]-rearrangements. Pudovik and Konovalova discovered that *α*-hydroxyphosphonates with certain structural requirements isomerize to phosphates under basic conditions and they named this reaction phosphonate–phosphate rearrangement [1–3] (Scheme 1). They and others extended it to *α*-mercapto- and *α*-aminophosphonates (X = S, NR) [1]. These isomerizations are reminiscent of the 1,2-Wittig [4] and 1,2-Brook rearrangement [5, 6]. When strong bases such as LDA, LiTMP, *n*-BuLi, or *s*-BuLi are used to deprotonate **4** to give **3**, the reverse process is induced. Then, the phosphorus atom migrates from the hetero- to the carbanionic carbon atom in **3**, a [1,2]-rearrangement as found by Sturtz and Corbel [7] and preparatively and mechanistically extensively studied by the group of Hammerschmidt [8–10].

**Scheme 2:**
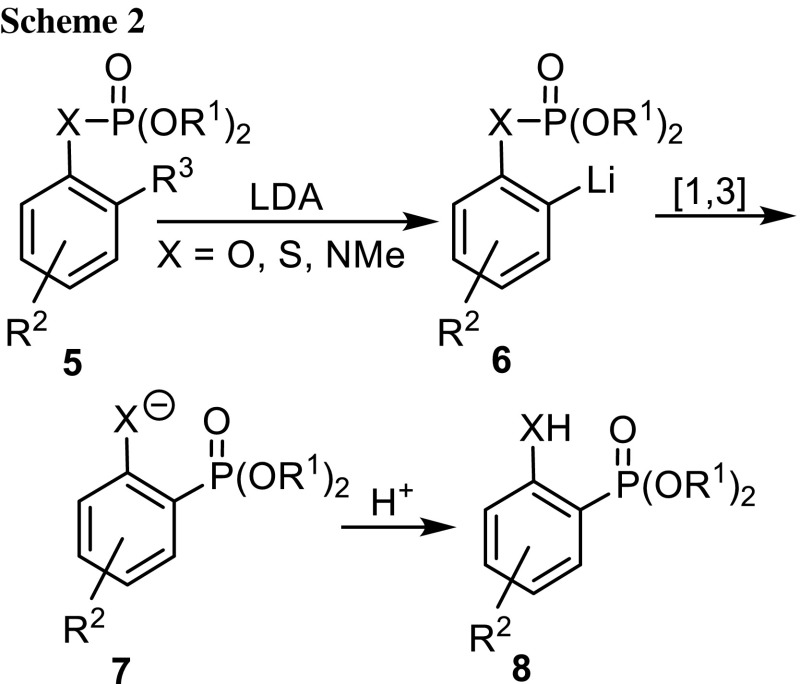
.

Aryl phosphates **5** (X = O) can be isomerized to *o*-hydroxyarylphosphonates **8** with strong bases [11–15] (Scheme 2). As the phosphate group effects directed *ortho*-metalation [16, 17], aryl lithiums **6** are formed, which undergo migration of the phosphorus atom from the oxygen to the carbanionic carbon atom to give phosphonates **7** and on workup **8**. The driving force for the rearrangement is the formation of the strong O–Li bond. A large variety of dialkyl aryl phosphates and derivatives thereof were rearranged. In analogy to the Fries-rearrangement this reaction was named anionic phospho-Fries rearrangement [11]. We prefer the more general term [1,3]-phosphate–hydroxyphosphonate rearrangement. The isomerization was applied to *S*-aryl thiophosphates and their derivatives [18–20]. Analogously, this isomerizations might be dubbed [1,3]-thiophosphate–sulfanylphosphonate rearrangement. Only one example for a [1,3]-phosphoramidate–aminophosphonate rearrangement (X = NMe) has been reported by Modro et al. [21]. Interestingly, they also found, that diphenyl *N*-methyl-*N*-phenylphosphoramidate underwent the first migration of phosphorus from the oxygen to the carbon atom when treated with 1 equiv of LDA, two migrations from O to C, when treated with 4 equiv of LDA and a third one from N to C with 8 equiv of LDA. We reasoned that *N*-arylphosphoramidates with an additional electron-withdrawing group on the nitrogen atom could first facilitate *o*-metalation of the phenyl ring and migration of the phosphorus atom from N to C and second expand the scope of the P to O to the P to N rearrangement.

## Results and discussion

**Scheme 3:**
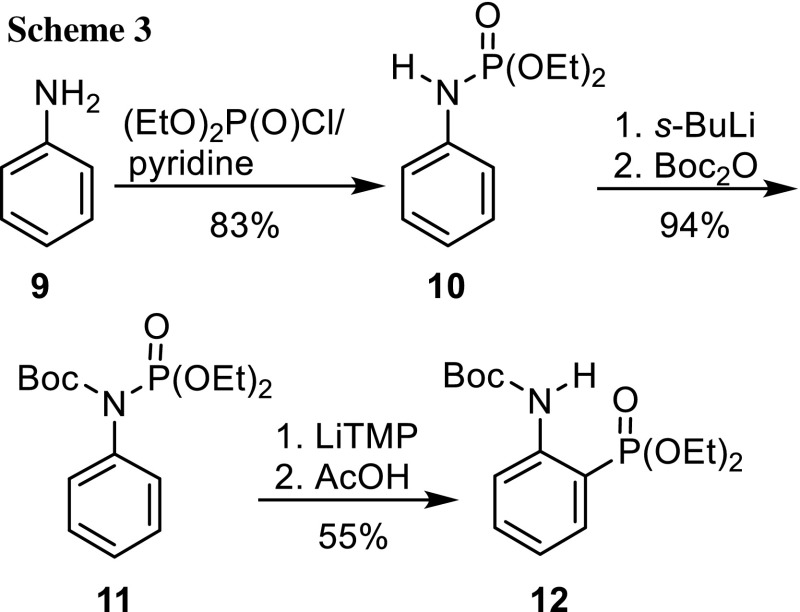
.

**Scheme 4:**
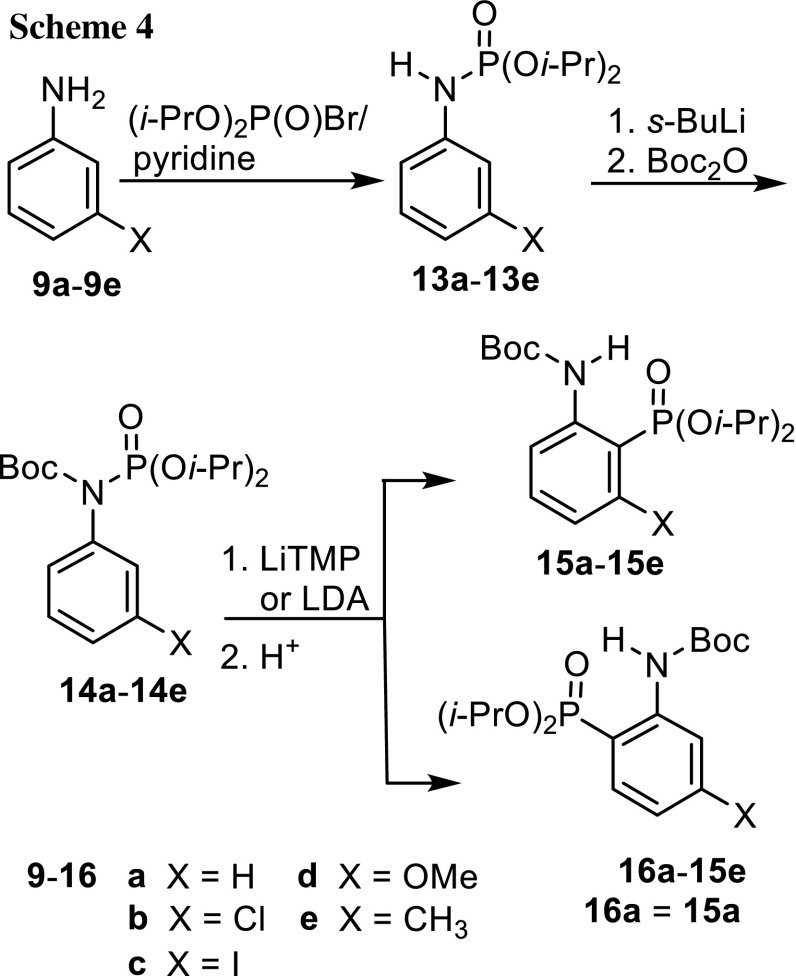
.

We found recently that dialkyl *N*-benzylphosphoramidates underwent a base-catalyzed [1,2]-phosphoramidate-*α*-aminophosphonate rearrangement when the secondary nitrogen atom was protected with a Boc or dialkoxyphosphoryl group [22, 23]. Importantly, both Boc and (RO)_2_P(O) at the nitrogen atom favor directed *o*-metalation [16, 17] with lithium bases. In order to test this idea, aniline was phosphorylated with diethyl chlorophosphate/pyridine to give *N*-phenylphosphoramidate **10** in 83% yield (Scheme 3). *N*-Boc protection was effected by deprotonation of **10** at the nitrogen atom with *s*-BuLi at −78 °C, followed by addition of Boc_2_O and allowing the reaction mixture to slowly warm to room temperature. Bases *n*-BuLi or *s*-BuLi were not used for metalation because of possible attack at the carbonyl group of the Boc group, which would regenerate phosphoramidate **10**. Therefore, we opted for strong encumbered amide bases such as lithium 2,2,6,6-tetramethylpiperidide (LiTMP) and LDA. When LiTMP was reacted with **11** in THF for 2 h at −78 °C, phosphonate **12** was obtained in 55% yield by flash chromatography. Surprisingly, even LiTMP removed the Boc group from part of **11** so that phosphoramidate **10** was isolated in 30% yield as well. LDA as alternative base in Et_2_O in combination with slowly warming the reaction mixture for 18 h furnished the same phosphonate in 62% yield and **10** as side product, too. These two experiments demonstrate that *N*-Boc-protected *N*-phenylphosphoramidate **11** can be rearranged by [1,3]-migration of phosphorus from the nitrogen to the carbon atom induced by strong lithium amide bases. Migration of the Boc group was not detected. We tested also **14a**, the isopropyl analog of **10**, as substrate for the rearrangement to optimize the reaction conditions (Scheme 4). It was prepared from aniline and diisopropyl bromophosphate [24] in 95% yield and then Boc-protected as before (92% yield). The rearrangement was performed in THF with three bases, *s*-BuLi/TMEDA, LDA, and LiTMP at −78 °C. The combination of *s*-BuLi with TMEDA delivered a mixture of recovered starting material **14a** and Boc-deprotected **13a** as crude product, which did not contain phosphonate **15a** unexpectedly. The other two bases gave the desired phosphonate **15a** in 25 and 79% yield, respectively. In summary, these results demonstrated that *N*-Boc-protected *N*-arylphosphoramidates can be isomerized to *N*-Boc-protected *o*-aminoarylphosphonates. As isopropyl protecting groups at the phosphorus atom seemed to shield it better than the ethyl groups and to give higher yields, all further experiments were performed with diisopropyl phosphoramidates.

In order to address the regioselectivity of metalation and expand the scope of the rearrangement, *meta*-substituted anilines **9b**–**9e** were transformed into *N*-Boc-protected phosphoramidates **14b**–**14e** by the previous methods in combined unoptimized yields ranging from 58 to 90% yield. All rearrangements were performed in THF with LiTMP as well as LDA, normally at −78 °C, sometimes for comparison reasons also at 0 °C, for 1 h in the majority of experiments (Table 1). Yields could possibly be increased by longer reaction times for certain substrates. Varying amounts of starting material **14** and the corresponding phosphoramidate **13** formed by base-catalyzed removal of Boc from **14** were present (TLC) in the crude reaction products, but were not isolated. In the case of the *N*-Boc-*N*-(*m*-chlorophenyl)phosphoramidate **14b** the two phosphonates **15b** and **16b** were formed in yields of 55 and 35% for LiTMP as base at −78 °C and 55 and 32% for the same base at 0 °C, respectively. LDA delivered exclusively **15b** in 96% yield, surprisingly. The preferred deprotonation *ortho* to both substituents might be explained by their additive acidifying and *o*-directing effect, although the bases were very encumbered. Consequently, the preferred formation of phosphonates **15b** is predetermined. When chloride as substituent was replaced by iodide being larger in size and lower in electronegativity compared to chloride, the quantity of phosphonate 1**6c** (60 and 66%, see Table 1) outweighed those of **15c** (19 and 23%) for LiTMP, independent of the reaction temperature. Remarkably, LDA delivered a yield of only 4% for **15c** but 78% for **16c**. The next two *N*-Boc-protected phosphoramidates, **14d** and **14e**, followed the expectations. The methoxy group with its strong *ortho* directing metalation effect in combination with the P=O group directed deprotonation exclusively to carbon atom 2, leading to the exclusive formation of phosphonate **15d**. However, the methyl group which reduces the acidity of the hydrogen atoms in the phenyl ring and is additionally of significant size effected that the rearrangement of **14e** furnished low yields of **16e** (5 and 6%), despite a reaction time of 20 h with slow warming to room temperature. The main components of the crude product were starting material **14e** and its precursor **13e** formed by base-catalyzed removal of Boc.

**Table 1:** [1,3]-Rearrangement of *N*-Boc-*N*-arylphosphoramidates **14b**–**14e** to *N*-Boc-protected *o*-aminoarylphosphonates **15b**–**15e** and **16b**–**16e** ^a^

**14**	Base	*T*/ °C	*t*/h	Yield/ % **15**/**16**
**b**	LiTMP	−78	1	55/35
**b**	LiTMP	0	1	55/32
**b**	LDA	−78	1	96/0
**c**	LiTMP	−78	1	19/60
**c**	LiTMP	0	1	23/66
**c**	LDA	−78	1	4/78
**d**	LiTMP	−78	1	40/0
**d**	LDA	−78	1	67/0
**e**	LiTMP	−78	20	0/5
**e**	LiTMP^b^	−78	1	0/6

^a^All reactions were performed in THF

^b^Two equiv of TMEDA were present as well

**Scheme 5:**
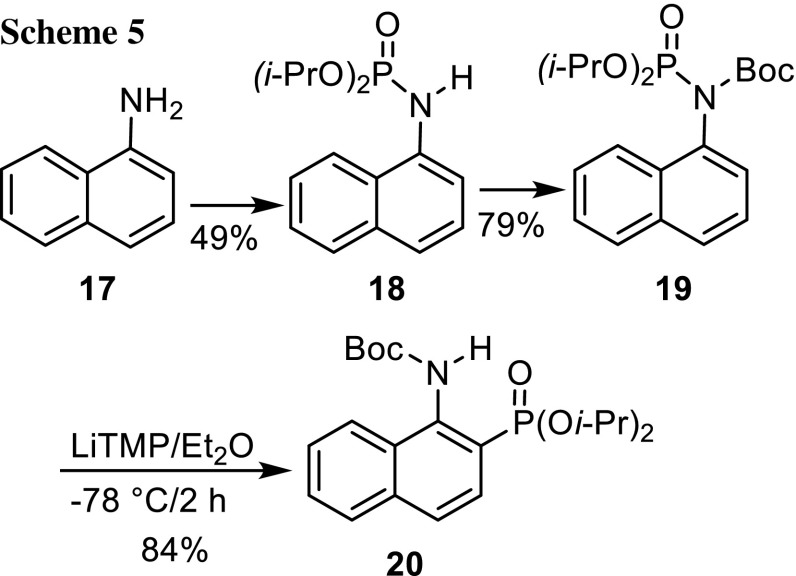
.

**Scheme 6:**
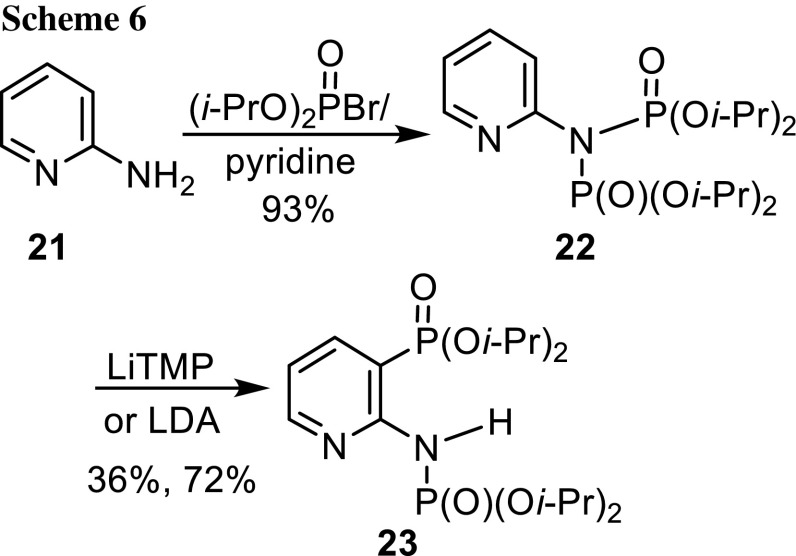
.

Two more amines were transformed into *N*-protected phosphoramidates. 1-Naphthylamine was phosphorylated and *N*-Boc protected in the usual way to give compound **19**, which was smoothly subjected to the [1,3]-phosphoramidate–aminophosphonate rearrangement with LiTMP in Et_2_O at −78 °C for 2 h (Scheme 5). The 2-napthylphosphonate **20** was isolated in 84% yield. The second amine was 2-aminopyridine (**21**), which was converted to diphosphoramidate **22** with 2 equiv of diisopropyl bromophosphate/excess pyridine in 72% yield in one step (Scheme 6). We found previously that two diethylphosphoryl groups at the nitrogen atom of benzyl amine allowed to perform a [1,2]-phosphoramidate-*α*-aminophosphonate rearrangement [22]. Here, LiTMP and LDA mediated a [1,3]-phosphoramidate-aminophosphonate rearrangement of diphosphoramidate **22** to phosphonate **23** in yields of 36 and 72%, respectively.

**Scheme 7:**
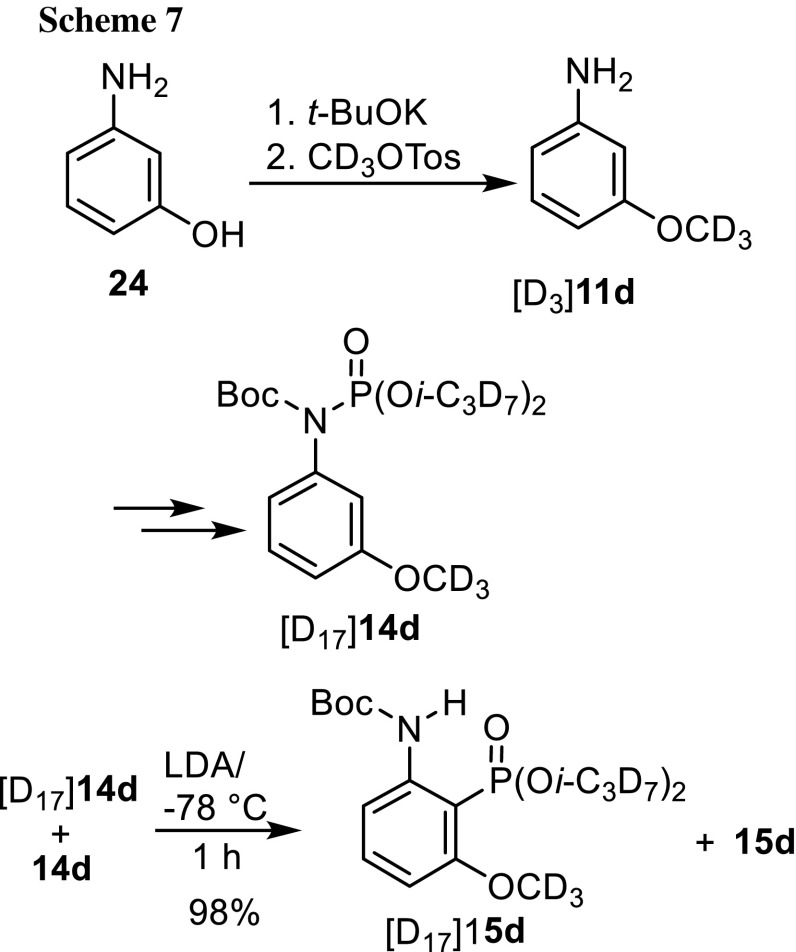
.

When we started this project we surmised that the migration of phosphorus from the nitrogen to the carbon atom is an intramolecular process proceeding via a cyclic four-membered transition state. As it is unfavorable, we reasoned that the formal [1,3]-migration could also be exclusively or in part an intermolecular process with phenyllithium attacking at the electrophilic phosphorus atom of a second molecule. We conceived a crossover experiment with an equimolar mixture of deuterated and nondeuterated *N*-Boc-*N*-(3-methoxyphenyl)phosphoramidate, [D_17_]**14d** and **14d** (Scheme 7). The latter was synthesized from [D_3_]methyl tosylate [25] and tris(heptadeuterioisopropyl) phosphite [24] by the methods used for the unlabeled species. The rearrangement of the 1:1 mixture furnished a 1:1 mixture of phosphonates **15d** and [D_17_]**15d** in 98% yield. No phosphonates containing 3 or 14 deuterium atoms could be detected by EI-MS, indicative of an intermolecular transfer of the phosphorus atom with its labeled substituents. Therefore, the migration of the phosphorus atom from the nitrogen to the *ortho*-carbon atom in *N*-Boc-*N*-arylphosphoramidates proceeds exclusively intramolecularly and represents a [1,3]-sigmatropic rearrangement. A modified crossover experiment was carried out by Heinicke et al. [26] demonstrating that aryl dialkyl phosphates isomerize to *o*-hydroxyphenylphosphonates intramolecularly.

**Scheme 8:**
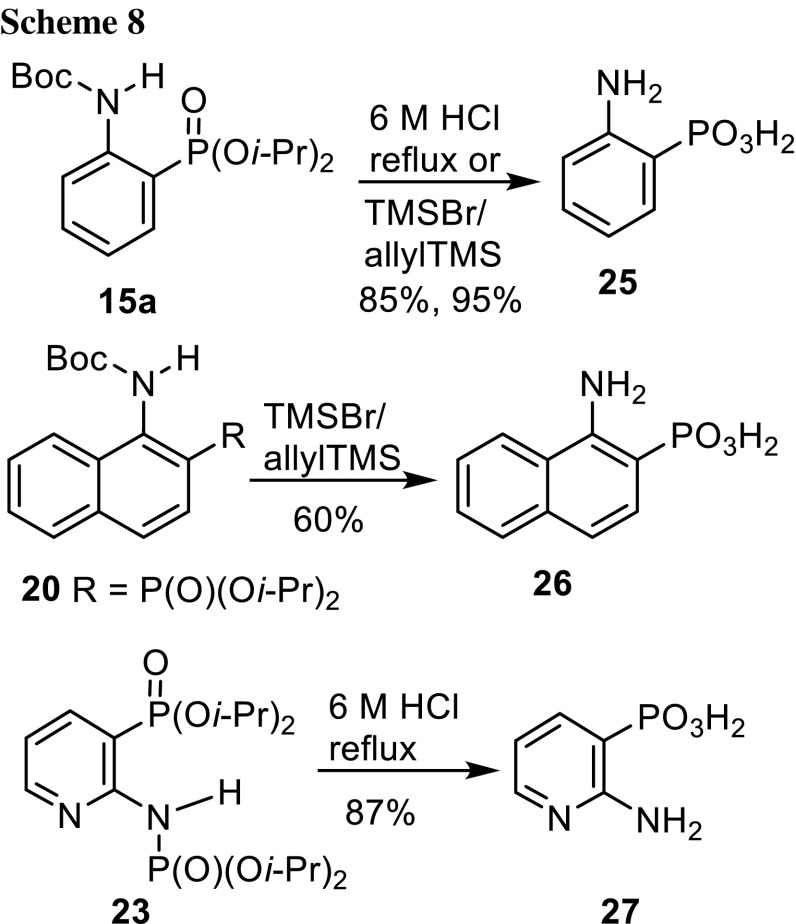
.

*o*-Aminophenylphosphonic acid was tested for its anticholesterinase activity [27] and 2-amino-4-methylphenylphosphonic acid for removing formaldehyde adducts [28] from RNA and DNA bases. The protected *o*-aminoarylphosphonates prepared by [1,3]-migration of phosphorus can be deprotected to give *o*-aminoarylphosphonic acids as shown for three examples (Scheme 8). *N*-Boc-*o*-aminophenylphosphonate **15a** was deprotected by refluxing with 6 M HCl or with TMSBr/allylTMS [29] in CH_2_Cl_2_ under milder conditions, resulting in a higher yield. The *o*-aminophenylphosphonic acid (**25**) was purified by cation exchange chromatography (Dowex 50 W × 8, H^+^) and crystallization from water. 2-Naphthylphosphonate **20** had to be deprotected with TMSBr/allylTMS and crystallized from methanol because of its lability in hot water, where it decomposed to 1-naphthylamine (detected by TLC) and H_3_PO_4_ (detected by ^31^P NMR). Pyridin-3-ylphosphonate **23** was converted to 2-amino-3-pyridin-3-ylphosphonic acid (**27**) using refluxing 6 M HCl, followed by purification as for **25**. Selective removal of the Boc group with CF_3_CO_2_H would give dialkyl *o*-aminophosphonates. As the amino group in aromatic compounds can be replaced by a variety of substituents, the globally or partially deprotected *N*-Boc-*o*-aminophoshonates prepared here are useful starting materials for other *o*-substituted phosphonic acid derivatives.

## Conclusion

In summary, we have shown that arylamines can be easily converted to *N*-Boc-*N*-arylphosphoramides. Treatment with LiTMP or LDA mediated [1,3]-phosphoramidate-aminophosphonate rearrangements. Three phosphonates were globally deprotected. This approach extends the scope for the synthesis of 2-amino-substituted phosphonic acids derived from aromatic and heteroaromatic amines. Additionally, selective removal of the Boc-protecting group will give a free amino group amenable to fuctional group manipulation and incorporation of the 2-aminophosphonates into peptides.

## Experimental

NMR pectra (^1^H, ^13^C, and ^31^P) were recorded in CDCl_3_ or D_2_O on a Bruker DRX 400 (^1^H: 400.13 MHz, ^13^C: 100.61 MHz, ^31^P: 161.98 MHz), spectrometer at 25 °C, respectively. Chemical shifts *δ* (ppm) were referenced to residual CHCl_3_ (*δ*_H_ = 7.24), CDCl_3_ (*δ*_C_ = 77.00), HDO (*δ*_H_ = 4.67), and external H_3_PO_4_ (85%; *δ*_P_ = 0.00). IR spectra of compounds soluble in CDCl_3_ or CH_2_Cl_2_ were recorded on a Perkin-Elmer FT 1600 IR Spectrometer. The solution was applied to a silicon disc [30] and the solvent was allowed to evaporate before the measurement. IR spectra of the aminophosphonic acids were recorded on a Perkin-Elmer Spectrum 2000 IR spectrometer in ATR mode. Melting points were determined on a Reichert Thermovar instrument. New compounds were checked for purity by means of appropriate combustion analysis results.

Flash (column) chromatography was performed with Merck silica gel 60 (230–400 mesh). Thin layer chromatography (TLC) was carried out on 0.25 mm thick Merck plates, silica gel 60 F_254_. Spots were visualized by UV and/or dipping the plate into a solution of (NH_4_)_6_Mo_7_O_24_·4H_2_O (24.0 g) and Ce(SO_4_)_2_·4H_2_O (1.0 g) in 10% aqueous H_2_SO_4_ (500 cm^3^), followed by heating with a heat gun. Pyridine was dried by refluxing over powdered CaH_2_, then distilled and stored over molecular sieves (4 Å). Dichloromethane was dried by storage over molecular sieves (3 Å). All other chemicals and solvents were of the highest purity available and used as received.

### General procedure A—*N*-phosphorylation of aromatic amines with diisopropyl bromophosphate

A solution of bromine (18 cm^3^, 18 mmol, 1 M in dry CH_2_Cl_2_) was added dropwise to a stirred solution of 4.44 cm^3^ (*i*-PrO)_3_P (3.75 g, 18 mmol) in 10 cm^3^ dry CH_2_Cl_2_ under Ar at −50 °C [24]. Stirring was continued for 30 min at 0 °C. After cooling again to −50 °C, a solution of aromatic amine (15 mmol) in 2 cm^3^ dry CH_2_Cl_2_ and 4.18 cm^3^ Et_3_N (3.04 g, 30 mmol) was added. The reaction mixture was allowed to warm to RT (18 h). HCl (12 cm^3^, 2 M) and 20 cm^3^ water were added. The organic layer was separated and the aqueous one was extracted twice with CH_2_Cl_2_. The combined organic layers were dried with Na_2_SO_4_ and concentrated under reduced pressure. The residue was purified by flash chromatography and/or crystallization.

### General procedure B—conversion of *N*-arylphosphoramidates to *N*-Boc-*N*-arylphosphoramidates

*s*-BuLi (5.14 cm^3^, 7.2 mmol, 1.4 M in cyclohexane) was added to a stirred solution of phosphoramidate (6 mmol) in 10 cm^3^ dry THF (or Et_2_O) under Ar at −78 °C. After 15 min a solution of 1.44 g Boc_2_O (6.6 mmol) in 5 cm^3^ dry THF (or Et_2_O) was added. The reaction mixture was allowed to slowly warm to RT in the cooling bath (18 h). After the addition of 3 cm^3^ solution of AcOH in Et_2_O (2 M) and 10 cm^3^ H_2_O the organic phase was separated and the aqueous one extracted with CH_2_Cl_2_ (3 × 10 cm^3^). The combined organic layers were washed with a saturated aqueous solution of NaHCO_3_, dried with Na_2_SO_4_, and concentrated under reduced pressure. The residue was purified by flash chromatography.

### General procedure C—rearrangement of *N*-Boc-*N*-arylphosphoramidates with lithium tetramethylpiperidide (LiTMP)

*n*-BuLi (0.94 cm^3^, 1.50 mmol, 1.6 M in hexane) was added to a stirred solution of 0.25 cm^3^ 2,2,6,6-tetramethylpiperidine (0.212 g, 1.5 mmol) in 8 cm^3^ dry THF (or Et_2_O) at 0 °C under Ar. Stirring was continued for 30 min at 0 °C. Subsequently, the mixture was cooled at −78 °C and a solution of *N*-Boc-*N*-arylphosphoramidate (1 mmol) dissolved in 2 cm^3^ dry THF (or Et_2_O) was added. After stirring for 2 h at −78 °C the reaction was quenched with 1 cm^3^ AcOH (2 M in Et_2_O) and 5 cm^3^ H_2_O. The organic layer was separated and the aqueous one was extracted twice with CH_2_Cl_2_. The combined organic layers were dried with Na_2_SO_4_ and concentrated under reduced pressure. The residue was purified by flash chromatography.

### General procedure D—rearrangement of *N*-Boc-*N*-arylphosphoramidates with LDA

*s*-BuLi (1.07 cm^3^, 1.5 mmol, 1.4 M in hexane) was added to a stirred solution of 0.21 cm^3^ diisopropylamine (0.152 g, 1.5 mmol) in 5 cm^3^ dry Et_2_O (or THF) at −20 °C under Ar. Stirring was continued for 20 min. Subsequently, a solution of *N*-Boc-*N*-arylphosphoramidate (1 mmol) dissolved in 2 cm^3^ dry Et_2_O was added. The stirred solution was allowed to slowly warm to RT (20 h) and then the reaction was quenched with 2 cm^3^ AcOH (2 M in Et_2_O) and 5 cm^3^ H_2_O. The organic layer was separated and the aqueous one was extracted twice with CH_2_Cl_2_. The combined organic layers were dried with Na_2_SO_4_ and concentrated under reduced pressure. The residue was purified by flash chromatography.

### Diethyl *N*-phenylphosphoramidate (**10**)

Diethyl chlorophosphate (3.451 g, 2.89 cm^3^, 20 mmol) was added to a stirred solution of 1.83 cm^3^ aniline (1.862 g, 20 mmol) and 3.35 cm^3^ Et_3_N (2.429 g, 24 mmol) in 36 cm^3^ dry CH_2_Cl_2_ under Ar at RT. After stirring for 19 h at RT the reaction was quenched with 20 cm^3^ H_2_O. The organic layer was separated and the aqueous one was extracted twice with CH_2_Cl_2_. The combined organic layers were dried with Na_2_SO_4_ and concentrated under reduced pressure. The residue was purified by crystallization from hexanes to give 3.79 g phosphoaramidate **10** (83%) as colorless crystals. M.p.: 93–94 °C (Ref. [31] 94–95 °C).

### Diethyl *N*-(*t*-butoxycarbonyl)-*N*-phenylphosphoramidate (**11**, C_15_H_24_NO_5_P)

Diethyl *N*-phenylphosphoramidate (**10**, 1.15 g, 5 mmol) was converted to the *N*-Boc derivative in THF and purified by flash chromatography (hexanes/EtOAc = 1/2, *R*_*f*_ = 0.45) to give 1.55 g *N*-Boc-phosphoramidate **11** (94%) as a colorless oil. ^1^H NMR (400.13 MHz, CDCl_3_): *δ* = 1.253 (dt, *J* = 1.0, 7.1 Hz, 3H), 1.251 (dt, *J* = 1.0, 7.1 Hz, 3H), 1.47 (br. s, 9H), 4.07 (m, 2H), 4.17 (m, 2H), 7.23 (m, 2H), 7.29 (m, 1H), 7.36 (m, 2H) ppm; ^13^C NMR (100.61 MHz, CDCl_3_): *δ* = 15.8 (d, *J* = 7.7 Hz, 2C), 27.8 (3C), 63.7 (d, *J* = 6.1 Hz, 2C), 82.6, 127.5 (d, *J* = 1.5 Hz), 128.7 (2C), 128.8 (d, *J* = 2.3 Hz, 2C), 138.1, 153.2 (d, *J* = 8.4 Hz) ppm; ^31^P NMR (161.98 MHz, CDCl_3_): *δ* = 0.2 ppm; IR (Si, CDCl_3_): $\bar{V}$ = 2982, 1728, 1492, 1369, 1302, 1160, 1099, 1028, 983 cm^−1^.

### Diethyl 2-(*t*-butoxycarbonylamino)phenylphosphonate (**12**, C_15_H_24_NO_5_P)

Diethyl *N*-Boc-*N*-phenylphosphoramidate (**11**, 0.329 g, 1 mmol) was rearranged according to general procedure C in THF and flash chromatogaphed (hexanes/EtOAc = 3/1, *R*_*f*_ = 0.45) to give 0.182 g 2-(*N*-Boc-amino)phenylphosphonate **12** (55%) as a colorless oil and 0.078 g phosphoramidate **10** (30%). When **11** (0.329 g, 1 mmol) was rearranged according to general procedure D in Et_2_O and flash chromatographed (hexanes/EtOAc = 3/1), the yield of phosphonate **12** was 0.203 g (62%). ^1^H NMR (400.13 MHz, CDCl_3_): *δ* = 1.30 (t, *J* = 7.1 Hz, 6H), 1.49 (s, 9H), 4.03 (m, 2H), 4.12 (m, 2H), 7.01 (ddt, *J* = 1.0, 3.0, 7.7 Hz, 1H), 7.47 (ddt, *J* = 1.5, 7.3, 7.7 Hz, 1H), 7.52 (ddd, *J* = 1.5, 7.7, 14.4 Hz, 1H), 8.32 (ddd, *J* = 1.0, 7.3. 7.8 Hz, 1H), 9.61 (br. s, 1H) ppm; ^13^C NMR (100.61 MHz, CDCl_3_): *δ* = 16.2 (d, *J* = 6.9 Hz, 2C), 28.3 (3C), 63.2 (d, *J* = 5.5 Hz, 2C), 80.4, 112.9 (d, *J* = 179.7 Hz), 119.2 (d, *J* = 11.5 Hz), 121.7 (d, *J* = 13.8 Hz), 132.6 (d, *J* = 6.1 Hz), 133.9 (d, *J* = 2.2 Hz), 143.4 (d, *J* = 7.7 Hz), 153.0 ppm; ^31^P NMR (161.98 MHz, CDCl_3_): *δ* = 34.1 ppm; IR (Si, CDCl_3_): $\bar{V}$ = 3247, 3117, 2981, 1732, 1587, 1538, 1443, 1305, 1243, 1160, 1094, 1044, 1022, 972 cm^−1^.

### Diisopropyl *N*-phenylphosphoramidate (**13a**, C_12_H_20_NO_3_P)

Aniline (1.40 g, 1.37 cm^3^, 15 mmol) was phosphorylated according to general procedure A and flash chromatographed (hexanes/EtOAc = 1/1, *R*_*f*_ = 0.68) to yield 3.65 g phosphoramidate **13a** (95%) as colorless crystals. M.p.: 120–121 °C (hexanes); ^1^H NMR (400.13 MHz, CDCl_3_): *δ* = 1.19 (d, *J* = 6.3 Hz, 6H), 1.36 (d, *J* = 6.3 Hz, 6H), 4.66 (dsept, *J* = 6.3, 7.6 Hz, 2H), 6.22 (br. s, 1H), 6.90 (tt, *J* = 1.0, 7.6 Hz, 1H), 6.97 (m, 2H), 7.20 (br. t, *J* = 7.6 Hz, 2H) ppm; ^13^C NMR (100.61 MHz, CDCl_3_): *δ* = 23.5 (d, *J* = 5.4 Hz, 2C), 23.9 (d, *J* = 4.6 Hz, 2C), 71.6 (d, *J* = 5.4 Hz, 2C), 117.4 (d, *J* = 7.7 Hz, 2C), 121.2, 129.0 (2C), 140.2 ppm; ^31^P NMR (161.98 MHz, CDCl_3_): *δ* = 1.3 ppm; IR (Si): $\bar{V}$ = 3161, 2981, 2919, 1604, 1504, 1426, 998, 963 cm^−1^.

### Diisopropyl *N*-(t-butoxycarbonyl)-*N*-phenylphosphoramidate (**14a**, C_17_H_28_NO_5_P)

Diisopropyl *N*-(phenyl)phosphoramidate (**13a**, 2.01 g, 7.81 mmol) was converted to the *N*-Boc derivative in 15 cm^3^ dry Et_2_O according to general procedure B. The crude product was purified by flash chromatography (CH_2_Cl_2_/EtOAc = 7/1, *R*_*f*_ = 0.40) to give 2.57 g *N*-Boc-*N*-phenylphosphoramidate **14a** (92%) as a colorless oil. ^1^H NMR (400.1 MHz, CDCl_3_): *δ* = 1.16 (d, *J* = 6.3 Hz, 6H), 1.31 (d, *J* = 6.3 Hz, 6H), 1.45 (s, 9H), 4.70 (dsept, *J* = 6.3, 7.3 Hz, 2H), 7.19 (m, 2H), 7.27 (m, 1H), 7.33 (m, 2H) ppm; ^13^C NMR (100.6 MHz, CDCl_3_): *δ* = 23.3 (d, *J* = 6.1 Hz, 2C), 23.9 (d, *J* = 3.8 Hz, 2C), 28.0 (3C), 72.8 (d, *J* = 6.1 Hz, 2C), 82.7, 127.5 (d, *J* = 1.5 Hz, 2C), 128.8, 129.1 (d, *J* = 2.3 Hz, 2C), 138.8, 153.6 ppm; ^31^P NMR (161.98 MHz, CDCl_3_): *δ* = 11.6 ppm; IR (Si, CDCl_3_): $\bar{V}$ = 2981, 1729, 1492, 1386, 1370, 1299, 1259, 1161, 1097, 1002 cm^−1^.

### Diisopropyl 2-(*t*-butoxycarbonylamino)phenylphosphonate (**15a**, C_17_H_28_NO_5_P)

Diisopropyl *N*-(*t*-butoxycarbonyl)-*N*-phenylphosphoramidate (**14a**, 0.514 g, 1.44 mmol) was converted to phenylphosphonate **15a** by general procedure C in THF. The crude product was purified by flash chromatography (hexanes/EtOAc = 5/1, *R*_*f*_ = 0.64) to give 0.404 g phosphonate **15a** (79%) as a colorless oil. Rearrangement of 0.514 g phosphoramidate **14a** (1.44 mmol) according to general procedure D in THF and flash chromatography (hexanes/EtOAc = 3/1, *R*_*f*_ = 0.60) of the crude product furnished 0.128 g phosphonate **15a** (25%). ^1^H NMR (400.13 MHz, CDCl_3_): *δ* = 1.20 (d, *J* = 6.3 Hz, 6H), 1.36 (d, *J* = 6.3 Hz, 6H), 1.49 (s, 9H), 4.64 (dsept, *J* = 6.3, 7.6 Hz, 2H), 7.00 (ddt, *J* = 1.0, 3.0, 7.7, 1H), 7.45 (ddt, *J* = 1.5, 7.3, 7.7 Hz, 1H), 7.55 (ddt, *J* = 1.5, 7.7, 14.6 Hz, 1H), 8.28 (ddd, *J* = 1.0, 7.3, 7.5 Hz, 1H), 9.56 (br. s, 1H) ppm; ^13^C NMR (100.63 MHz, CDCl_3_): *δ* = 23.7 (d, *J* = 4.6 Hz, 2C), 24.0 (d, *J* = 3.8 Hz, 2C), 28.3 (3C), 71.4 (d, *J* = 5.4 Hz, 2C), 80.3, 114.8 (d, *J* = 181.3 Hz), 119.2 (d, *J* = 11.5 Hz), 121.6 (d, *J* = 13.8 Hz), 132.8 (d, *J* = 6.1 Hz), 133.6 (d, *J* = 2.3 Hz), 142.8 (d, *J* = 6.9 Hz), 153.0 ppm; ^31^P NMR (161.98 MHz, CDCl_3_): *δ* = 31.9 ppm; IR (Si, CDCl_3_): $\bar{V}$ = 3247, 1732, 1587, 1305, 1243, 1160, 985 cm^−1^.

### Diisopropyl *N*-(3-chlorophenyl)phosphoramidate (**13b**, C_12_H_19_ClNO_3_P)

3-Chloroaniline (1.021 g, 0.84 cm^3^, 8 mmol) was converted to (3-chlorophenyl)phosphoramidate **13b** according to general procedure A. The crude product was purified by flash chromatography (hexanes/EtOAc = 2/1, *R*_*f*_ = 0.18) to give 2.22 g phosphoramidate **13b** (95%) as colorless crystals. M.p.: 121–122 °C (hexanes); ^1^H NMR (400.13 MHz, CDCl_3_): *δ* = 1.21 (d, *J* = 6.3 Hz, 6H), 1.37 (d, *J* = 6.3 Hz, 6H), 4.66 (dsept, *J* = 6.3, 7.6 Hz, 2H), 6.87 (m, 2H), 6.90 (br. s, 1H), 7.01 (t, *J* = 2.0 Hz, 1H), 7.11 (t, *J* = 8.1 Hz, 1H) ppm; ^13^C NMR (100.61 MHz, CDCl_3_): *δ* = 23.5 (d, *J* = 5.4 Hz, 2C), 23.8 (d, *J* = 4.6 Hz, 2C), 71.9 (d, *J* = 5.4 Hz, 2C), 115.5 (d, *J* = 7.6 Hz), 117.4 (d, *J* = 7.6 Hz), 121.2, 130.0, 134.7, 141.7 ppm; ^31^P NMR (161.98 MHz, CDCl_3_): *δ* = 0.8 ppm; IR (Si): $\bar{V}$ = 3153, 2980, 1599, 1498, 1481, 1388, 1225, 995 cm^−1^.

### Diisopropyl *N*-(*t*-butoxycarbonyl)-*N*-(3-chlorophenyl)phosphoramidate (**14b**, C_17_H_27_ClNO_5_P)

Diisopropyl *N*-(3-chlorophenyl)phosphoramidate (**13b**, 1.46 g, 5 mmol) was converted to *N*-Boc derivative **14b** in Et_2_O, using general procedure B. The crude product was purified by flash chromatography (hexanes/EtOAc = 1/1, *R*_*f*_ = 0.48) to give 1.36 g *N*-Boc-phosphormadiate **14b** (69%) as colorless crystals. M.p.: 80 °C (hexanes); ^1^H NMR (400.13 MHz, CDCl_3_): *δ* = 1.21 (d, *J* = 6.3 Hz, 6H), 1.33 (d, *J* = 6.3 Hz, 6H), 1.45 (s, 9H), 4.71 (dsept, *J* = 6.3, 7.1 Hz, 2H), 7.11 (m, 1H), 7.21 (m, 1H), 7.26 (m, 2H) ppm; ^13^C NMR (100.65 MHz, CDCl_3_): *δ* = 23.4 (d, *J* = 5.4 Hz, 2C), 23.9 (d, *J* = 4.6 Hz, 2C), 28.0 (3C), 73.0 (d, *J* = 6.1 Hz, 2C), 83.1, 127.5 (d, *J* = 2.3 Hz), 127.8, 129.5 (d, *J* = 2.3 Hz), 129.7, 134.1, 139.9, 153.2 (d, *J* = 7.7 Hz) ppm; ^31^P NMR (161.98 MHz, CDCl_3_): *δ* = −2.4 ppm; IR (Si, CDCl_3_): $\bar{V}$ = 2981, 1729, 1302, 1267, 1159, 1000 cm^−1^.

### Diisopropyl (2-*t*-butoxycarbonylamino-6-chlorophenyl)phosphonate and diisopropyl (2-*t*-butoxycarbonylamino-4-chlorophenyl)phosphonate (**15b** and **16b**, C_17_H_27_ClNO_5_P)

*N*-(*t*-Butoxycarbonyl)-*N*-(3-chlorophenyl)phosphoramidate (**14b**, 0.392 g, 1 mmol) was rearranged in THF according to general procedure C for 1 h at −78 °C. The crude product was purified by flash chromatography (hexanes/EtOAc = 5/1, **15b**: *R*_*f*_ = 0.70, **16b**: *R*_*f*_ = 0.60) to give 0.216 g phosphonate **15b** (55%) as colorless crystals, m.p.: 70–75 °C (hexanes), and 0.137 g phosphonate **16b** (35%) as colorless oil. When the same experiment was performed according to general procedure C for 1 h at 0 °C, 0.215 g **15b** (55%) and 0.125 g **16b** (32%) were obtained. When 392 mg *N*-(*t*-butoxycarbonyl)-*N*-(3-chlorophenyl)phosphoramidate (**14b**, 1 mmol) was rearranged according to general procdure D in THF for 1 h at −78 °C, 0.377 g phosphonate **15b** (96%) was obtained.

**15b**: ^1^H NMR (400.13 MHz, CDCl_3_): *δ* = 1.23 (d, *J* = 6.3 Hz, 6H), 1.38 (d, *J* = 6.3 Hz, 6H), 1.50 (s, 9H), 4.68 (dsept, *J* = 6.3, 7.8 Hz, 2H), 7.01 (ddd, *J* = 1.0, 4.3, 7.8 Hz, 1H), 7.33 (br. t, *J* = 8.3 Hz, 1H), 8.41 (ddd, *J* = 1.0, 5.6, 8.6 Hz, 1H), 11.11 (s, 1H) ppm; ^13^C NMR (100.61 MHz, CDCl_3_): *δ* = 23.5 (d, *J* = 4.6 Hz, 2C), 23.9 (d, *J* = 4.6 Hz, 2C), 28.3 (3C), 72.2 (d, *J* = 6.1 Hz, 2C), 80.4, 112.1 (d, *J* = 179.7 Hz), 117.2 (d, *J* = 10.7 Hz), 124.0 (d, *J* = 9.9 Hz), 133.7 (d, *J* = 1.5 Hz), 137.9, 146.3 (d, *J* = 6.1 Hz), 153.2 ppm; ^31^P NMR (161.98 MHz, CDCl_3_): *δ* = 15.4 ppm; IR (Si, CDCl_3_): $\bar{V}$ = 2981, 1732, 1582, 1444, 1397, 1298, 1269, 1239, 1160, 1124, 1061, 995 cm^−1^.

**16b**: ^1^H NMR (400.13 MHz, CDCl_3_): *δ* = 1.18 (d, *J* = 6.1 Hz, 6H), 1.33 (d, *J* = 6.1 Hz, 6H), 1.47 (s, 9H), 4.61 (dsept, *J* = 6.1, 7.8 Hz, 2H), 6.96 (dt, *J* = 1.8, 8.3 Hz, 1H), 7.43 (dd, *J* = 8.3, 14.4 Hz, 1H), 8.40 (dd, *J* = 1.8, 5.8 Hz, 1H), 9.71 (br.s, 1H) ppm; ^13^C NMR (100.61 MHz, CDCl_3_): *δ* = 23.6 (d, *J* = 5.4 Hz, 2C), 23.9 (d, *J* = 3.8 Hz, 2C), 28.2 (3C), 71.6 (d, *J* = 5.4 Hz, 2C), 80.8, 112.8 (d, *J* = 183.6 Hz), 118.9 (d, *J* = 11.5 Hz), 121.8 (d, *J* = 14.5 Hz), 133.8 (d, *J* = 6.9 Hz), 139.9 (d, *J* = 3.8 Hz), 143.9 (d, *J* = 8.4 Hz), 152.6 ppm; ^31^P NMR (162.0 MHz, CDCl_3_): *δ* = 17.7 ppm; IR (Si, CDCl_3_): $\bar{V}$ = 3233, 2981, 1732, 1599, 1577, 1520, 1411, 1275, 1240, 1160, 1077, 987 cm^−1^.

### Diisopropyl *N*-(3-iodophenyl)phosphoramidate (**13c**, C_12_H_19_INO_3_P)

Iodoaniline (1.752 g, 0.95 cm^3^, 8 mmol) was converted to *N*-(2-iodophenyl)phosphoramidate **13c**. The crude product was purified by flash chromatography (hexanes/EtOAc = 1/1, *R*_*f*_ = 0.51) to give 2.60 g phosphoramidate **13c** (85%) as colorless crystals. M.p.: 136–137 °C (hexanes); ^1^H NMR (400.13 MHz, CDCl_3_): *δ* = 1.22 (d, *J* = 6.3 Hz, 6H), 1.37 (d, *J* = 6.3 Hz, 6H), 4.67 (dsept, *J* = 6.3, 7.6 Hz, 2H), 6.24 (br. d, *J* = 8.8 Hz, 1H), 6.90 (dt, *J* = 1.8, 7.8 Hz, 1H), 7.03 (dt, *J* = 1.8, 7.8 Hz, 1H), 7.06 (t, *J* = 7.8 Hz, 1H), 7.14 (t, *J* = 1.8 Hz, 1H) ppm; ^13^C NMR (100.61 MHz, CDCl_3_): *δ* = 23.6 (d, *J* = 5.4 Hz, 2C), 23.8 (d, *J* = 4.6 Hz, 2C), 72.0 (d, *J* = 4.6 Hz, 2C), 116.0 (d, *J* = 7.7 Hz), 120.3 (d, *J* = 7.7 Hz), 122.8, 124.3, 130.4, 141.6 ppm; ^31^P NMR (161.98 MHz, CDCl_3_): *δ* = 0.4 ppm; IR (Si): $\bar{V}$ = 3148, 2979, 1581, 1480, 1220, 994, 974 cm^−1^.

### Diisopropyl *N*-(*t*-butoxycarbonyl)-*N*-(3-iodophenyl)phosphoramidate (**14c**, C_17_H_27_INO_5_P)

*n*-BuLi (1.63 cm^3^, 2.6 mmol, 1.6 *M* in hexane) was added to a stirred solution of 0.44 cm^3^ TMP (0.367 g, 2.6 mmol) in 4.5 cm^3^ dry THF at 0 °C under argon. After stirring for 15 min at 0 °C a solution of 0.39 cm^3^ TMEDA (0.302 g, 2.6 mmol) was added and stirring was continued for 15 min. Then, the mixture was cooled at −78 °C and a solution of 0.990 g *N*-(3-iodophenyl)phosphoramidate **13c** (2.6 mmol) dissolved in 2 cm^3^ dry THF was added, followed 15 min later by 0.567 g Boc_2_O (2.6 mmol) dissolved in 2 cm^3^ dry THF. The stirred solution was allowed to warm to RT (20 h) and then the reaction was quenched with 1.5 cm^3^ AcOH (2 M in dry Et_2_O) and 5 cm^3^ H_2_O. The organic layer was separated and the aqueous one was extracted twice with CH_2_Cl_2_. The combined organic layers were dried with Na_2_SO_4_ and concentrated under reduced pressure. The residue was purified by flash chromatography (CH_2_Cl_2_/EtOAc = 7/1, *R*_*f*_ = 0.31) to give 0.850 g *N*-Boc-phospharamidate **14c** (68%) as colorless crystals. M.p.: 75–77 °C (hexanes); ^1^H NMR (400.13 MHz, CDCl_3_): *δ* = 1.20 (d, *J* = 6.3 Hz, 6H), 1.32 (d, *J* = 6.3 Hz, 6H), 1.45 (s, 9H), 4.71 (dsept, *J* = 6.3, 7.1 Hz, 2H), 7.06 (t, *J* = 7.8 Hz, 1H), 7.18 (ddt, *J* = 1.0, 1.7, 7.8 Hz, 1H), 7.55 (dt, *J* = 1.0, 1.7 Hz, 1H), 7.61(dq, *J* = 1.0, 7.8 Hz, 1H) ppm; ^13^C NMR (100.61 MHz, CDCl_3_): *δ* = 23.4 (d, *J* = 6.1 Hz, 2C), 23.9 (d, *J* = 3.8 Hz, 2C), 28.0 (3C), 73.1 (d, *J* = 6.1 Hz, 2C), 83.1, 93.3, 128.6 (d, *J* = 2.3 Hz), 130.2, 136.6, 138.1 (d, *J* = 2.3 Hz), 139.9, 153.2 (d, *J* = 7.6 Hz) ppm; ^31^P NMR (161.98 MHz, CDCl_3_): *δ* = −2.4 ppm; IR (Si): $\bar{V}$ = 2980, 2930, 1727, 1300, 1159, 1098, 1002 cm^−1^.

### Diisopropyl (2-*t*-butoxycarbonylamino-6-iodophenyl)- and diisopropyl (2-*t*-butoxycarbonylamino-4-iodophenyl)phosphonate (**15c** and **16c**, C_17_H_27_INO_5_P)

*N*-(*t*-Butoxycarbonyl)-*N*-(3-iodophenyl)phosphoramidate (**14c**, 0.483 g, 1.0 mmol) was rearranged according to general procedure C in THF at −78 °C for 1 h. The crude product was purified by flash chromatography (hexanes/EtOAc = 5/1, **15c**: *R*_*f*_ = 0.62, **16c**: *R*_*f*_ = 0.53) to give 0.093 g **15c** (19%) as colorless crystals, m.p.: 75–77 °C (hexanes), and 0.288 g **16c** (60%) as a colorless oil. When this experiment was repeated except that the rearrangement was perfomed at 0 °C for 1 h, 0.112 g **15c** (23%) and 0.319 g **16c** (66%) were obtained. When 0.483 g *N*-Boc-phosphoramidate **14c** (1 mmol) was rearranged according to general procedure D in THF at −78 °C for 1 h, 0.019 g **15c** (4%) and 0.376 g **16c** (78%) were obtained.

**15c**: ^1^H NMR (400.13 MHz, CDCl_3_): *δ* = 1.28 (d, *J* = 6.3 Hz, 6H), 1.41 (d, *J* = 6.3 Hz, 6H), 1.49 (s, 9H), 4.71 (dsept, *J* = 6.3, 7.3 Hz, 2H), 7.01 (dd = ~t, *J* = 7.8, 8.6 Hz, 1H), 7.66 (ddd, *J* = 1.0, 3.4, 7.8 Hz, 1H), 8.48 (ddd, *J* = 1.0, 5.6, 8.6 Hz, 1H), 11.16 (br. s, 1H) ppm; ^13^C NMR (100.61 MHz, CDCl_3_): *δ* = 23.7 (d, *J* = 4.6 Hz, 2C), 24.0 (d, *J* = 4.6 Hz, 2C), 28.4 (3C), 72.5 (d, *J* = 4.4 Hz, 2C), 80.4, 99.1 (d, *J* = 1.5 Hz), 116.8 (d, *J* = 182.8 Hz), 118.8 (d, *J* = 10.7), 133.8 (d, *J* = 2.3 Hz), 135.9 (d, *J* = 11.5 Hz), 146.4 (d, *J* = 7.7 Hz), 153.2 ppm; ^31^P NMR (161.98 MHz, CDCl_3_): *δ* = 15.1 ppm; IR (Si, CDCl_3_): $\bar{V}$ = 2980, 1731, 1577, 1437, 1388, 1252, 1160, 1116, 1059, 993 cm^−1^.

**16c**: ^1^H NMR (400.13 MHz, CDCl_3_): *δ* = 1.19 (d, *J* = 6.3 Hz, 6H), 1.35 (d, *J* = 6.3 Hz, 6H), 1.49 (s, 9H), 4.62 (dsept, *J* = 6.3, 7.8 Hz, 2H), 7.13 (~dt, *J* = 2.0, 8.3, 1H), 7.37 (dd, *J* = 8.3, 14.4, 1H), 8.57 (dd, *J* = 1.5, 5.8, 1H), 9.70 (bs, 1H, NH) ppm; ^13^C NMR (100.61 MHz, CDCl_3_): *δ* = 24.1 (d, *J* = 5.4 Hz, 2C), 24.4 (d, *J* = 3.8 Hz, 2C), 28.7 (3C), 71.7 (d, *J* = 5.4 Hz, 2C), 80.8, 113.3 (d, *J* = 183.6 Hz), 121.8 (d, *J* = 11.5 Hz), 124.7 (d, *J* = 14.5 Hz), 128.5 (d, *J* = 3.8 Hz), 133.8 (d, *J* = 6.9 Hz), 143.8 (d, *J* = 7.7 Hz), 152.7 ppm; ^31^P NMR (161.98 MHz, CDCl_3_): *δ* = 17.8 ppm; IR (Si, CDCl_3_): $\bar{V}$ = 3233, 2980, 1731, 1594, 1575, 1408, 1274, 1238, 1160, 1073, 987 cm^−1^.

### Diisopropyl *N*-(3-methoxyphenyl)phosphoramidate (**13d**, C_13_H_22_NO_4_P)

3-Methoxyaniline (0.985 g, 0.90 cm^3^, 8 mmol) was converted to phosphoramidate **13d** according to general procedure A. The crude product was purified by flash chromatography (CH_2_Cl_2_/EtOAc = 10/1, *R*_*f*_ = 0.08) to give 2.21 g **13d** (96%) as colorless crystals. M.p.: 139–141 °C (hexanes); ^1^H NMR (400.13 MHz, CDCl_3_): *δ* = 1.22 (d, *J* = 6.3 Hz, 6H), 1.36 (d, *J* = 6.3 Hz, 6H), 3.75 (s, 3H), 4.66 (dsept, *J* = 6.3, 7.6 Hz, 2H), 5.55 (br. d, *J* = 9.1 Hz, 1H), 6.47 (ddd, *J* = 1.0, 2.0, 8.0 Hz, 1H), 6.53 (m, 2H), 7.10 (t, *J* = 8.1 Hz, 1H) ppm; ^13^C NMR (100.61 MHz, CDCl_3_): *δ* = 23.6 (d, *J* = 5.4 Hz, 2C), 23.9 (d, *J* = 4.6 Hz, 2C), 55.2, 71.8 (d, *J* = 4.6 Hz, 2C), 103.4 (d, *J* = 7.7 Hz), 106.9, 110.0 (d, *J* = 7.7 Hz), 129.9, 141.2, 160.4 ppm; ^31^P NMR (161.98 MHz, CDCl_3_): *δ* = 0.7 ppm; IR (Si): $\bar{V}$ = 3169, 2979, 1608, 1488, 1408, 1277, 1233, 1161, 1054, 982 cm^−1^.

### Diisopropyl *N*-(*t*-butoxycarbonyl)-*N*-(3-methoxyphenyl)phosphoramidate (**14d**, C_18_H_30_NO_6_P)

Diisopropyl *N*-(3-methoxyphenyl)phosphoramidate (**13d**, 1.436 g, 5 mmol) was converted to *N*-Boc derivative **14d** by general procedure B in Et_2_O at −78 °C. The crude product was flash chromatographed (EtOAc, *R*_*f*_ = 0.60) to give 1.78 g **14d** (92%) as a colorless oil. ^1^H NMR (400.13 MHz, CDCl_3_): *δ* = 1.17 (d, *J* = 6.3 Hz, 6H), 1.32 (d, *J* = 6.3 Hz, 6H), 1.46 (s, 9H), 3.77 (s, 3H), 4.70 (dsept, *J* = 6.3, 7.6 Hz, 2H), 6.75 (m, 1H), 6.81 (m, 2H), 7.23 (t, *J* = 8.1 Hz, 1H) ppm; ^13^C NMR (100.61 MHz, CDCl_3_): *δ* = 23.3 (d, *J* = 6.1 Hz, 2C), 23.9 (d, *J* = 3.8 Hz, 2C), 28.0 (3C), 55.3, 72.9 (d, *J* = 6.1 Hz, 2C), 82.8, 113.3, 115.1 (d, *J* = 2.3 Hz), 121.5 (d, *J* = 1.5 Hz), 129.4, 139.7 (d, *J* = 2.3 Hz), 153.5 (d, *J* = 8.4 Hz), 159.9 ppm; ^31^P NMR (161.98 MHz, CDCl_3_): *δ* = −1.9 ppm; IR (Si, CDCl_3_): $\bar{V}$ = 2981, 1729, 1605, 1492, 1370, 1302, 1257, 1163, 1097, 1008 cm^−1^.

### Diisopropyl (2-*t*-butoxycarbonylamino-6-methoxyphenyl)phosphonate (**15d**, C_18_H_30_NO_6_P)

*N*-(*t*-Butoxycarbonyl)-*N*-(3-methoxyphenyl)phosphoramidate (**14d**, 0.387 g, 1 mmol) was rearranged by general procedure C in THF at −78 °C for 1 h. The crude product was flash chromatographed (hexanes/EtOAc = 5/1, *R*_*f*_ = 0.61) to give 0.156 g **15d** (40%) as colorless crystals, m.p.: 85–87 °C (hexanes). When 0.387 g **14d** (1 mmol) was rearranged by general procedure D in THF at −78 °C for 1 h 0.260 g phosphonate **15d** (67%) was obtained. ^1^H NMR (400.13 MHz, CDCl_3_): *δ* = 1.15 (d, *J* = 6.1 Hz, 6H), 1.34 (d, *J* = 6.1 Hz, 6H), 1.49 (s, 9H), 3.80 (s, 3H), 4.59 (dsept, *J* = 6.1, 8.3 Hz, 2H), 6.50 (ddd, *J* = 0.8, 5.3, 8.4 Hz, 1H), 7.38 (t, *J* = 8.4 Hz, 1H), 8.06 (ddd, *J* = 0.8, 5.8, 8.4 Hz, 1H), 11.00 (s, 1H) ppm; ^13^C NMR (100.61 MHz, CDCl_3_): *δ* = 23.5 (d, *J* = 4.6 Hz, 2C), 24.0 (d, *J* = 4.6 Hz, 2C), 28.4 (3C), 55.5, 71.0 (d, *J* = 5.4 Hz), 79.9, 101.8 (d, *J* = 175.2 Hz), 104.1(d, *J* = 8.4 Hz), 111.3 (d, *J* = 11.5 Hz), 134.6, 145.7 (d, *J* = 5.4 Hz), 153.3, 162.2 ppm; ^31^P NMR (161.98 MHz, CDCl_3_): *δ* = 17.9 ppm; IR (Si, CDCl_3_): $\bar{V}$ = 3172, 3085, 2980, 1729, 1603, 1586, 1469, 1413, 1268, 1241, 1204, 1161, 1046, 995 cm^−1^.

### Diisopropyl *N*-(3-methylphenyl)phosphoramidate (**13e**, C_13_H_22_NO_3_P)

*m*-Toluidine (0.857 g, 0.86 cm^3^, 8 mmol) was converted to phosphoramidate **13e** by general procedure A. The crude product was flash chromatographed (CH_2_Cl_2_/EtOAc = 1/1, *R*_*f*_ = 0.56) and gave 1.98 g **13e** (91%) as colorless crystals. M.p.: 90–93 °C (hexanes); ^1^H NMR (400.13 MHz, CDCl_3_): *δ* = 1.20 (d, *J* = 6.3 Hz, 6H), 1.36 (d, *J* = 6.3 Hz, 6H), 2.27 (s, 3H), 4.65 (dsept, *J* = 6.3, 7.8 Hz, 2H), 5.68 (br. d, *J* = 9.1 Hz, 1H), 6.73 (d, *J* = 7.3 Hz, 1H), 6.76 (s, 1H), 6.77 (d, *J* = 7.3 Hz, 1H), 7.09 (t, *J* = 7.3 Hz, 1H) ppm; ^13^C NMR (100.61 MHz, CDCl_3_): *δ* = 21.5, 23.6 (d, *J* = 5.4 Hz, 2C), 23.9 (d, *J* = 3.8 Hz, 2C), 71.6 (d, *J* = 4.6 Hz, 2C), 114.4 (d, *J* = 6.9 Hz), 118.1 (d, *J* = 7.4 Hz), 122.2, 128.9, 139.0, 139.9 ppm; ^31^P NMR (161.98 MHz, CDCl_3_): *δ* = 1.2 ppm; IR (Si): $\bar{V}$ = 3172, 2980, 1610, 1595, 1487, 1387, 1231, 1176, 990 cm^−1^.

### Diisopropyl-*N*-(*t*-butoxycarbonyl)-*N*-(3-methylphenyl)phosphoramidate (**14e**, C_18_H_30_NO_5_P)

Diisopropyl *N*-(3-methylphenyl)phosphoramidate (**13e**, 1.36 g, 5 mmol) was converted to *N*-Boc phosphoramdiate **14e** by general procedure B in Et_2_O. The crude product was flash chromatographed (hexanes/EtOAc = 1/1, *R*_*f*_ = 0.55) and delivered 1.69 g **14e** (91%) as colorless crystals. M.p.: 54–55 °C (hexanes); ^1^H NMR (400.13 MHz, CDCl_3_): *δ* = 1.17 (d, *J* = 6.3 Hz, 6H), 1.32 (d, *J* = 6.3 Hz, 6H), 1.46 (s, 9H), 2.32 (s, 3H), 4.69 (dsept, *J* = 6.3, 7.1 Hz, 2H), 6.99 (br. d, *J* = 7.6 Hz, 1H), 7.00 (br. s, 1H), 7.07 (br. d, *J* = 7.6 Hz, 1H), 7.21 (t, *J* = 7.6 Hz, 1H) ppm; ^13^C NMR (100.61 MHz, CDCl_3_): *δ* = 21.2, 23.3 (d, *J* = 6.1 Hz, 2C), 23.9 (d, *J* = 3.8 Hz, 2C), 28.1 (3C), 72.7 (d, *J* = 5.4 Hz, 2C), 82.6, 126.0 (d, *J* = 1.5 Hz), 128.4, 128.6, 129.7 (d, *J* = 2.3 Hz), 138.6 (d, *J* = 2.3 Hz), 138.7, 153.7 ppm; ^31^P NMR (161.98 MHz, CDCl_3_): *δ* = −1.7 ppm; IR (Si, CDCl_3_): $\bar{V}$ = 2981, 1729, 1386, 1369, 1300, 1256, 1160, 1098, 1001 cm^−1^.

### Diisopropyl (2-*t*-butoxycarbonylamino-4-methylphenyl)phosphonate (**16e**, C_18_H_30_NO_5_P)

Diisopropyl *N*-(*t*-butoxycarbonyl)-*N*-(3-methylphenyl)phosphoramidate (**14e**, 0.743 g, 2 mmol) was rearranged to *N*-Boc phosphonate **16e** according to general procedure C in THF for 5 h. The crude product, which contained a large amount of starting material, was flash chromatographed (hexanes/EtOAc = 7/1, *R*_*f*_ = 0.27) and furnished 0.038 g **16e** (5%) as a colorless oil. When this experiment (1 mmol) was repeated, except that 0.3 cm^3^ TMEDA (2 mmol) was added before cooling to −78 °C and that the reaction mixture was allowed to warm to RT within 5 h, 0.024 g *N*-Boc phosphonate **16e** (6%) was obtained as colorless oil. ^1^H NMR (400.13 MHz, CDCl_3_): *δ* = 1.18 (d, *J* = 6.3 Hz, 6H), 1.34 (d, *J* = 6.3 Hz, 6H), 1.49 (s, 9H), 2.33 (s, 3H), 4.60 (dsept, *J* = 6.3, 7.8 Hz, 2H), 6.81 (br. d, *J* = 7.8 Hz, 1H), 7.42 (dd, *J* = 7.8 Hz, 1H), 8.12 (d, *J* = 6.3 Hz, 1H), 9.55 (s, 1H) ppm; ^13^C NMR (100.61 MHz, CDCl_3_): *δ* = 22.0, 23.6 (d, *J* = 5.4 Hz, 2C), 24.0 (d, *J* = 3.8 Hz, 2C), 28.3 (3C), 71.2 (d, *J* = 5.4 Hz, 2C), 80.2, 111.6 (d, *J* = 184.3 Hz), 120.9 (d, *J* = 11.5 Hz), 122.6 (d, *J* = 14.5 Hz), 132.7 (d, *J* = 6.9 Hz), 142.7 (d, *J* = 7.6 Hz), 144.5 (d, *J* = 2.3 Hz), 153.0 ppm; ^31^P NMR (161.98 MHz, CDCl_3_): *δ* = 19.1 ppm; IR (Si, CDCl_3_): $\bar{V}$ = 3247, 2980, 1731, 1580, 1244, 1162, 1089, 985 cm^−1^.

### Diisopropyl *N*-(1-naphthyl)phosphoramidate (**18**, C_16_H_22_NO_3_P)

1-Naphthylamine (1.146 g, 8.0 mmol) was converted to phosphoramdiate **18** by general procedure A. The crude product was purified by flash chromatography (hexanes/EtOAc = 2/1, *R*_*f*_ = 0.30) and delivered 1.21 g **18** (49%) as colorless crystals. M.p.: 149 °C (hexanes); ^1^H NMR (400.13 MHz, CDCl_3_): *δ* = 1.18 (d, *J* = 6.1 Hz, 6H), 1.38 (d, *J* = 6.1 Hz, 6H), 4.71 (dsept, *J* = 6.1, 7.6 Hz, 2H), 5.71 (d, *J* = 7.8 Hz, 1H), 7.36 (m, 2H), 7.49 (m, 3H), 7.82 (m, 1H), 7.90 (m, 1H) ppm; ^13^C NMR (100.61 MHz, CDCl_3_): *δ* = 23.6 (d, *J* = 5.4 Hz, 2C), 23.9 (d, *J* = 3.8 Hz, 2C), 71.9 (d, *J* = 5.4 Hz, 2C), 114.0 (d, *J* = 2.3 Hz), 120.0, 122.3, 125.1 (d, *J* = 9.9 Hz), 125.9, 125.9, 126.0, 128.8, 134.3, 135.0 ppm; ^31^P NMR (161.98 MHz, CDCl_3_): *δ* = 1.5 ppm; IR (Si): $\bar{V}$ = 2979, 1470, 1244, 991 cm^−1^.

### Diisopropyl *N*-(*t*-butoxycarbonyl)-*N*-(1-naphthyl)phosphoramidate (**19**, C_21_H_30_NO_5_P)

Diisopropyl *N*-(1-naphthyl)phosphoramidate (**18**, 0.922 g, 3.0 mmol) was converted to Boc derivative **19** by general procedure B in Et_2_O. The crude product was purified by flash chromatography (CH_2_Cl_2_/EtOAc = 10/1, *R*_*f*_ = 0.35) and gave 0.960 g **19** (79%) as brownish oil. ^1^H NMR (400.13 MHz, CDCl_3_): *δ* = 0.89 (d, *J* = 6.1 Hz, 3H), 1.11 (d, *J* = 6.1 Hz, 3H), 1.25 (d, *J* = 6.1 Hz, 3H), 1.28 (d, *J* = 6.1 Hz, 3H), 1.40 (s, 9H), 4.62 (dsept, *J* = 6.1, 7.1 Hz, 1H), 4.72 (dsept, *J* = 6.1, 7.1 Hz, 1H), 7.38 (td, *J* = 7.3, 1.5 Hz, 1H), 7.44 (dd, *J* = 7.7, 7.3 Hz, 1H), 7.46 (ddd, *J* = 8.1, 6.8, 1.3 Hz, 1H), 7.52 (ddd, *J* = 8.3, 6.8, 1.5 Hz, 1H), 7.79 (br. d, *J* = 8.3 Hz, 1H), 7.82 (br. d, *J* = 7.7 Hz, 1H), 8.02 (br. d, *J* = 8.6 Hz, 1H) ppm; ^13^C NMR (100.61 MHz, CDCl_3_): *δ* = 22.9 (d, *J* = 6.9 Hz), 23.3 (d, *J* = 6.9 Hz), 23.8 (d, *J* = 3.1 Hz), 24.0 (d, *J* = 3.1 Hz), 27.9 (3C), 73.0 (d, *J* = 6.1 Hz), 73.1 (d, *J* = 6.1 Hz), 82.6, 123.0, 125.4, 126.0, 126.6 (d, *J* = 3.1 Hz), 126.7, 128.1, 128.3 (d, *J* = 1.5 Hz), 131.7, 134.3, 135.4, 153.6 (d, *J* = 9.2 Hz) ppm; ^31^P NMR (161.98 MHz, CDCl_3_): *δ* = −1.5 ppm; IR (Si, CDCl_3_): $\bar{V}$ = 2980, 1727, 1301, 1159, 1002 cm^−1^.

### Diisopropyl (1-*t*-butoxycarbonylamino-2-naphthyl)phosphonate (**20**, C_21_H_30_NO_5_P)

Diisopropyl *N*-(*t*-butoxycarbonyl)-*N*-(1-naphthyl)phosphoramidate (**19**, 0.469 g, 1.15 mmol) was rearranged to *N*-Boc phosphonate **20** according to general procedure C in Et_2_O at −78 °C for 2 h. The crude product was flash chromatographed (hexanes/EtOAc = 2/1, *R*_*f*_ = 0.54) and gave 0.393 g **20** (84%) as a colorless oil. ^1^H NMR (400.13 MHz, CDCl_3_): *δ* = 1.21 (d, *J* = 6.1 Hz, 6H), 1.38 (d, *J* = 6.1 Hz, 6H), 1.50 (s, 9H), 4.72 (dsept, *J* = 6.1, 8.1 Hz, 2H), 7.53 (m, 2H), 7.76 (m, 3H), 8.0 (m, 1H), 8.17 (br. s, 1H) ppm; ^13^C NMR (100.61 MHz, CDCl_3_): *δ* = 23.8 (d, *J* = 4.6 Hz, 2C), 24.0 (d, *J* = 3.8 Hz, 2C), 28.3 (s, 3C), 71.4 (d, *J* = 5.4 Hz, 2C), 80.4, 119.7 (d, *J* = 183.6 Hz), 125.8, 126.0, 126.3, 127.1 (d, *J* = 7.7 Hz), 127.8, 128.1, 129.3 (d, *J* = 13.0 Hz), 136.3, 139.2 (d, *J* = 5.4 Hz), 154.0 ppm; ^31^P NMR (161.98 MHz, CDCl_3_): *δ* = 17.0 ppm; IR (Si, CDCl_3_): $\bar{V}$ = 3401, 3271, 2979, 2930, 1733, 1243, 1161, 986 cm^−1^.

### Tetraisopropyl *N*-(pyridin-2-yl)diphosphoramidate (**22**, C_17_H_32_N_2_O_6_P_2_)

2-Aminopyridine (0.753 g, 8 mmol) was converted to diphosphoramidate **22** by general procedure A except that 19.2 mmol bromine and 19.2 mmol (*i*-PrO)_3_P, 32 mmol Et_3_N and 3 cm^3^ 2 N HCl were used. The crude product was purified by flash chromatography (EtOAc/EtOH = 10/1, *R*_*f*_ = 0.55) to give 3.13 g **22** (93%) as a colorless oil. ^1^H NMR (400.13 MHz, CDCl_3_): *δ* = 1.23 (d, *J* = 6.3 Hz, 12H), 1.30 (d, *J* = 6.3 Hz, 12H), 4.83 (m, 4H), 7.16 (m, 1H), 7.32 (br. d, *J* = 7.8 Hz, 1H), 7.66 (dt, *J* = 1.8, 7.8 Hz, 1H), 8.46 (dd, *J* = 1.8, 4.8 Hz, 1H) ppm; ^13^C NMR (100.61 MHz, CDCl_3_): *δ* = 23.4 (d, *J* = 3.2 Hz, 2C), 23.4 (d, *J* = 3.1 Hz, 2C), 23.8 (dd, *J* = 2.3 Hz, 2C), 23.8 (d, *J* = 1.5 Hz, 2C), 72.8 (d, *J* = 3.2 Hz, 2C), 72.8 (dd, *J* = 3.1 Hz, 2C), 122.2, 123.2 (t, *J* = 2.7 Hz), 138.0, 148.9, 152.3 ppm; ^31^P NMR (161.98 MHz, CDCl_3_): *δ* = −1.5 ppm; IR (Si): $\bar{V}$ = 2980, 2936, 1589, 1467, 1433, 1386, 1375, 1279, 1179, 1143, 1109, 1000 cm^−1^.

### Diisopropyl (2-(diisopropylphosphorylamino)pyridin-3-yl)phosphonate (**23**, C_17_H_32_N_2_O_6_P_2_)

Tetraisopropyl *N*-(pyridin-2-yl)diphosphoramidate (**22**, 1.27 g, 3 mmol) was rearranged to pyridin-3-ylphosphonate **23** by general procedure D in THF for 20 h. The crude product was flash chromatographed (CH_2_Cl_2_/EtOH = 20/1, *R*_*f*_ = 0.38) to give 0.920 g **23** (72%) as an oil. When this experiment was repeated with LiTMP according to general procedure C, 0.460 g **23** (36%) was obtained. ^1^H NMR (400.13 MHz, CDCl_3_): *δ* = 1.20 (d, *J* = 6.1 Hz, 6H), 1.30 (d, *J* = 6.1 Hz, 6H), 1.34 (d, *J* = 6.1 Hz, 6H), 1.36 (d, *J* = 6.1 Hz, 6H), 4.65 (dsept, *J* = 6.1 Hz, 7.8 Hz, 2H), 4.83 (dsept, *J* = 6.1, 7.8 Hz, 2H), 6.83 (ddd, *J* = 2.0, 4.8, 7.3 Hz, 1H), 7.76 (ddt, *J* = 2.0, 7.3, 14.9 Hz, 1H), 8.40 (dt, *J* = 2.0, 4.8 Hz, 1H), 8.56 (br. d, *J* = 10.9 Hz, 1H) ppm; ^13^C NMR (100.61 MHz, CDCl_3_): *δ* = 23.6 (d, *J* = 5.4 Hz, 2C), 23.7 (d, *J* = 4.6 Hz, 2C), 23.9 (d, *J* = 4.6 Hz, 2C), 24.0 (d, *J* = 4.6 Hz, 2C), 71.7 (d, *J* = 5.4 Hz, 2C), 72.1 (d, *J* = 6.1 Hz, 2C), 108.8 (dd, *J* = 10.3, 185.9 Hz), 115.8 (d, *J* = 9.9 Hz), 142.1 (d, *J* = 6.9 Hz), 152.4, 156.1 (d, *J* = 10.7 Hz) ppm; ^31^P NMR (161.98 MHz, CDCl_3_): *δ* = −1.1 (d, *J* = 2.0 Hz), 16.9 (d, *J* = 2.0 Hz) ppm; IR (Si, CDCl_3_): $\bar{V}$ = 3218, 2980, 2935, 1575, 1445, 1262, 1231, 998 cm^−1^.

### Crossover experiment

#### *3*-*[Methyl*-*D*_*3*_*]methoxyaniline* ([D_3_]**9d**, C_7_H_6_D_3_NO)

To a stirred mixture of 0.546 g *3*-aminophenol (5 mmol) and 0.786 g *t*-BuOK (7 mmol) in 8 cm^3^ dry THF under argon at RT was added 1.136 g [D_3_]methyl *p*-toluenesulfonate (6 mmol) [25] dropwise. After stirring for 18 h 5 cm^3^ H_2_O and CH_2_Cl_2_ were added. The organic phase was separated and the aqueous one was extracted with CH_2_Cl_2_. The combined organic layers were dried with Na_2_SO_4_ and concentrated under reduced pressure. The residue was purified by flash chromatography (CH_2_Cl_2_, *R*_*f*_ = 0 42) to give 0.400 g labeled *3*-methoxyaniline [D_3_]**9d** (63%) as a colorless oil. ^1^H NMR (250.13 MHz, CDCl_3_): *δ* = 3.63 (br. s, 2H), 6.22 (d, *J* = 2.2 Hz, 1H), 6.28 (ddd, *J* = 0.9, 2.2, 8.0 Hz, 1H), 7.04 (t, *J* = 8.0 Hz, 1H).

##### Di-[D_7_]isopropyl *N*-(3-[methyl-D_3_]methoxyphenyl)phosphoramidate ([D_17_]**13d**, C_13_H_5_D_17_NO_4_P)

3-[Methyl-D_3_]methoxyaniline (0.630 g, 5 mmol) was converted to 1.28 g phosphoramidate [D_17_]**13d** (84%) as colorless crystals by the method used for the unlabled compound except that (*i*-PrO)_3_P was replaced by the deuterated species [24]; m.p.: 141–143 °C (hexanes). The ^1^H and ^13^C NMR spectrum of [D_17_]**13d** were identical to those of the unlabeled compound except for the missing signals because of deuteration.

##### Di-[D_7_]isopropyl *N*-(*t*-butoxycarbonyl)-*N*-(3-[methyl-D_3_]methoxyphenyl)phosphoramidate ([D_17_]**14d**, C_18_H_13_D_17_NO_6_P)

Di-[D_7_]isopropyl *N*-(3-[methyl-D_3_]methoxyphenyl)phosphoramidate ([D_17_]**13d**) (0.609 g, 2 mmol) was converted to 0.760 g *N*-Boc phosphoramidate [D_17_]**14d** (94%) as a colorless oil. The ^1^H and ^13^C NMR spectrum of [D_17_]**14d** were identical to those of the unlabeled compound except for the missing signals because of deuteration. ^31^P NMR (161.98 MHz, CDCl_3_): *δ* = −1.9 ppm; IR (Si, CDCl_3_): $\bar{V}$ = 2980, 1728, 1604, 1491, 1289, 1156, 1112, 990, 914 cm^−1^.

##### Crossover experiment with a 1:1 mixture of deuterated and nondeuterated diisopropyl *N*-(*t*-butoxycarbonyl)-*N*-(3-methoxyphenyl)phosphoramidate

A mixture of 0.202 g deuterated *N*-(*t*-butoxycarbonyl)-*N*-(3-methoxyphenyl)phosphoramidate [D_17_]**14d** (0.5 mmol) and 0.194 g nondeuterated *N*-(3-methoxyphenyl)-*N*-(*t*-butoxycarbonyl)phosphoramidate **14d** (0.5 mmol) was rearranged in the same way (LDA, THF, 1 h, 78 °C) as phosphoramidate **14d** and gave 0.390 g 1:1 mixture of deuterated phosphonate [D_17_]**15d** and nondeuterated **15d** (98%) as colorless crystals. M.p.: 87–89 °C (hexanes); ^1^H NMR (400.13 MHz, CDCl_3_): *δ* = 1.15 (d, *J* = 6.1 Hz, 6H), 1.34 (d, *J* = 6.1 Hz, 6H), 1.49 (s, 18H), 3.81 (s, 3H), 4.60 (dsept, *J* = 6.1, 8.3 Hz, 2H), 6.50 (dddd, *J* = 0.8, 2.8, 5.3, 8.3 Hz, 2H), 7.38 (t, *J* = 8.6, 2H), 8.06 (dd, *J* = 5.8, 8.6, 2H), 11.01 (br. s, 1H) ppm; ^31^P NMR (161.98 MHz, CDCl_3_): *δ* = 17.88 (P(O)(O*i*-Pr)_2_), 17.93 (br. s, P(O)(OCD(CD_3_)_2_)_2_) ppm; IR (Si, CDCl_3_): $\bar{V}$ = 3503, 3172, 3085, 2980, 2933, 1729, 1603, 1586, 1534, 1469, 1439, 1413, 1387, 1368, 1309, 1268, 1241, 1204, 1161, 1127, 1109, 1066, 1046, 995 cm^−1^. MS before rearrangement: EI-MS (70 eV, 50 °C): *m/z* (%) = 404 (6.3, MD_17_^+^), 387 (5.5, M^+^), 203 (94), 208 (100); after rearrangement: EI-MS (70 eV, 50 °C): *m/z* (%) = 404 (23.7, MD_17_^+^), 387 (25.3, M^+^), 203 (100), 208 (94). There were no signals with *m/z* = 401 or 390.

##### 2-Aminophenylphosphonic acid (**25**)

Method A: A mixture of 0.335 g diisopropyl 2-(*t*-butoxycarbonylamino)phenylphosphonate (**15a**, 0.94 mmol) and 10 cm^3^ HCl (6 M) was refluxed for 7 h. The cooled solution was concentrated under reduced pressure and the residue was dried over KOH in a vacuum desiccator. Then, it was dissolved in H_2_O and applied to an ion exchange column (Dowex 50 W × 8; H^+^) and eluted with H_2_O. Fractions containing product (TLC: *i*-PrOH/H_2_O/NH_3_ = 6/3/1, *R*_*f*_ = 0.38) were pooled and concentrated to give 0.138 g phosphonic acid **24** (85%) as a brownish crystalline product containing an impurity (6%, by ^1^ NMR); m.p.: 186 °C (water) [Ref. [27] 199–200 °C (water/ethanol)].

Method B: A solution of 0.530 g diisopropyl 2-(*t*-butoxycarbonylamino)phenylphosphonate (**15a**, 1.48 mmol), 0.48 cm^3^ allyltrimethylsilane (0.343 g, 3 mmol), and 1.73 cm^3^ bromotrimethylsilane (2.050 g, 13.4 mmol) [29] in 5 cm^3^ dry CH_2_Cl_2_ was refluxed under Ar for 20 h. Volatiles were removed under reduced pressure (0.5 mbar), CH_2_Cl_2_ (2 × 10 cm^3^) was added and removed again under reduced pressure. Water (15 cm^3^) was added and the reaction mixture was stirred for 15 min. The solvent was removed under reduced pressure and the residue was dried. Crystallization of the crude product from H_2_O gave 0.240 g **24** (95%) of brownish crystals, m.p.: 198–201 °C.

^1^H NMR (400.13 MHz, D_2_O/NaOD): *δ* = 6.70 (dd, *J* = 4.8, 7.6 Hz, 1H), 6.73 (br. t, *J* = 7.6 Hz, 1H), 7.13 (t, *J* = 7.6 Hz, 1H), 7.49 (dd, *J* = 7.6, 12.9 Hz, 1H) ppm; ^13^C NMR (100.63 MHz, D_2_O/NaOD): *δ* = 117.3 (d, *J* = 10.0 Hz), 118.6 (d, *J* = 12.2 Hz), 125.3 (d, *J* = 162.9 Hz), 130.7, 132.8 (d, *J* = 6.9 Hz), 147.8 (d, *J* = 5.4 Hz) ppm; ^31^P NMR (161.98 MHz, D_2_O/NaOD): *δ* = 11.7 ppm; IR (ATR): $\bar{V}$ = 2325, 1580, 1538, 1483, 1446, 1168, 1148, 1124, 1074, 1007, 903 cm^−1^.

##### *1*-*Amino*-*2*-*naphthylphosphonic acid* (**26**, C_10_H_10_NO_3_P)

Diisopropyl [1-*N*-(*t*-butoxycarbonylamino)-2-naphthyl]phosphonate (**20**, 0.618 g, 1.52 mmol) was deblocked by method B used for the preparation of **25** for the phenyl analog, except that the final procedure was different. After removing all volatiles under reduced pressure the residue was dissolved in 90 cm^3^ MeOH. Slow concentration of the reddish-brown solution on the rotary evaporator at RT to about 7 cm^3^ gave reddish crystals, which were collected by centrifugation. They were washed with 3 cm^3^ MeOH and dried for 1 h (50 °C/0.5 mbar) to give 0.204 g analytically pure naphthylphosphonic acid **26** (60%); m.p.: 236–239 °C; from the mother liquor a second crop (0.085 g, 95% pure by ^31^P NMR) was obtained. 1-Amino-2-naphthylphosphonic acid seemed to be stable for short periods of time in refluxing MeOH. It could not be crystallized from hot H_2_O, as it decomposed to phosphoric acid (by ^31^P NMR) and 1-naphthylamine (by TLC after extraction) within 30 min. ^1^H NMR (400.13 MHz, D_2_O/NaOD): *δ* = 7.20 (m, 1H), 7.37 (m, 1H), 7.56 (m, 1H), 7.69 (m, 2H), 7.86 (m, 1H) ppm; ^13^C NMR (100.61 MHz, D_2_O/NaOD): *δ* = 118.0 (d, *J* = 12.2 Hz), 120.0 (d, *J* = 164.5 Hz), 122.2, 124.6 (d, *J* = 10.7 Hz), 125.7, 127.0, 128.5, 130.3 (d, *J* = 7.7 Hz), 134.6, 143.7 (d, *J* = 6.1 Hz) ppm; ^31^P NMR (161.98 MHz, D_2_O/NaOD): *δ* = 12.5 ppm; IR (ATR): $\bar{V}$ = 2547, 1586, 1559, 1506, 1462, 1432, 1373, 1352, 1173, 1110, 1005, 944 cm^−1^.

##### *2*-*Aminopyridin*-*3*-*ylphosphonic acid* (**27**, C_5_H_7_N_2_O_3_P)

Diisopropyl 2-(diisopropylphosphorylamino)pyridin-3-ylphosphonate (**23**, 0.887 g, 2.10 mmol) was deblocked with refluxing HCl by method A used for the preparation of **25**. Fractions containing product (TLC: *i*-PrOH/H_2_O/NH_3_ = 6/3/1, *R*_*f*_ = 0.43) were pooled and concentrated to give 0.319 g **27** (87%) as nearly colorless crystals. M. p.: 256–259 °C (H_2_O/EtOH); ^1^H NMR (400.13 MHz, D_2_O): *δ* = 6.72 (ddd, *J* = 1.7, 6.3, 7.1 Hz, 1H), 7.60 (dt, *J* = 1.7, 6.3 Hz, 1H), 7.99 (ddd, *J* = 1.7, 7.1, 13.1 Hz, 1H) ppm; ^13^C NMR (100.61 MHz, D_2_O): *δ* = 130.1 (d, *J* = 11.5 Hz), 123.7 (d, *J* = 163.7 Hz), 136.6, 146.9 (d, *J* = 5.4 Hz), 154.4 (d, *J* = 12.2 Hz) ppm; ^31^P NMR (161.98 MHz, D_2_O): *δ* = 5.9 ppm; IR (ATR): $\bar{V}$ = 3401, 3297, 3079, 2627, 2325, 1650, 1615, 1593, 1554, 1456, 1401, 1334, 1244, 1169, 1144, 1011, 908 cm^−1^.
